# Selenium and Selenoproteins in Gut Inflammation—A Review

**DOI:** 10.3390/antiox7030036

**Published:** 2018-03-01

**Authors:** Shaneice K. Nettleford, K. Sandeep Prabhu

**Affiliations:** 1 Center for Molecular Immunology and Infectious Disease and Center for Molecular Toxicology and Carcinogenesis, The Pennsylvania State University, University Park, PA 16802, USA; sjn5208@psu.edu; 2 Department of Veterinary and Biomedical Sciences, The Pennsylvania State University, University Park, PA 16802, USA

**Keywords:** IBD, NF-κB, PPARγ, immune cells, innate lymphoid cells

## Abstract

Inflammatory bowel disease (IBD), characterized by severe flares and remissions, is a debilitating condition. While the etiology is unknown, many immune cells, such as macrophages, T cells and innate lymphoid cells, are implicated in the pathogenesis of the disease. Previous studies have shown the ability of micronutrient selenium (Se) and selenoproteins to impact inflammatory signaling pathways implicated in the pathogenesis of the disease. In particular, two transcription factors, nuclear factor-κB (NF-κB), and peroxisome proliferator activated receptor (PPAR)γ, which are involved in the activation of immune cells, and are also implicated in various stages of inflammation and resolution, respectively, are impacted by Se status. Available therapies for IBD produce detrimental side effects, resulting in the need for alternative therapies. Here, we review the current understanding of the role of NF-κB and PPARγ in the activation of immune cells during IBD, and how Se and selenoproteins modulate effective resolution of inflammation to be considered as a promising alternative to treat IBD.

## 1. Introduction

Inflammatory Bowel Disease (IBD), is a generalized term that encompasses Crohn’s disease and ulcerative colitis. Characterized by relapses and remissions, IBD patients experience debilitating symptoms varying from abdominal pain to rectal bleeding and anemia [1]. While ulcerative colitis is restricted to the colon, Crohn’s disease affects any portion of the gastrointestinal tract [2]. IBDs result from dysregulated immune responses to the intestinal microbiota due to genetic predispositions [2,3]. The emerging evidence suggests that there is a genetic component, in addition to environmental influences and microbial factors along with immune responses involved in the pathogenesis of these inflammatory disorders [3]. The dysbiosis of the microbiota contributes to the pathogenesis of IBD. There is a decrease in the diversity of the microbiota during IBD, especially anaerobic bacteria including, but not limited to, *Lactobacillus*, *Escherichia* and *Bacteroides* [4]. As it pertains to immune responses, transcription factors and nuclear receptors including, but not limited to, nuclear factor-kappa B, NF-κB, and peroxisome proliferator-activated receptor gamma, PPARγ, have been implicated in the pathogenesis of IBDs [4,5]. 

Currently, IBDs are incurable. Treatments for the diseases are aimed at alleviating the debilitating symptoms to ensure long term remission. To treat inflammation, a characteristic of IBDs, anti-inflammatory steroids and immunosuppressants are often used [1]. In extreme cases, resection of parts of the bowel is performed as an alternative means of treatment [1]. However, these anti-inflammatory and immunosuppressive agents are somewhat ineffective in a percentage (20%) of the patients who use them and can result in deleterious side effects associated with infections arising from the inhibition of the immune system [6,7] that is accompanied by poor resolution or wound healing. Thus, there is a need for new therapeutics that lack any detrimental side effects, while enhancing the pathways of resolution and immune regulatory networks mitigating inflammation. 

Selenium (Se) is an essential micronutrient that exists in the form of selenocysteine (Sec), the 21st amino acid, upon its incorporation into selenoproteins via the tRNA^[Sec]^ encoded by *Trsp* [8]. A few epidemiological studies indicate Se levels in patients of both ulcerative colitis and Crohn’s disease to be reduced [9,10]. There is a decrease in selenoprotein P (SEPP1) in the serum, as well as decrease in the activity of glutathione peroxidase in Crohn’s Disease patients [9,11]. Similarly, selenoprotein S (SelenoS) and selenoprotein K (SelenoK) have been implicated in inflammation and IBD [12,13,14]. In fact, it has been reported that there is an increase in the production of inflammatory cytokines, with a decrease in the expression of SelenoS [13]. Interestingly, in the absence of SelenoK, the opposite is observed, with a decrease in inflammatory cytokines. These differences could be context-dependent and need to be further investigated. Along with a decrease in Se levels, there is an increase in prostaglandin E_2_ (PGE_2_) in the plasma of patients with ulcerative colitis [15]. A few experimental models of IBD and associated colon cancer suggest Se and selenoproteins to play a key role in inflammatory tumorigenesis and inflammatory microenvironment [16,17]. Previous studies from our laboratory has reported that Se, in the form of selenoproteins, can shunt the arachidonic acid pathway from the production of more pro-inflammatory mediators such as PGE_2_ and interleukin (IL)-1β to more anti-inflammatory mediators such as prostaglandin D_2_ (PGD_2_) and its cyclopentenone metabolites, ∆^12^-prostaglandin J_2_ (∆^12^-PGJ_2_) and 15-deoxy-∆^12,14^-prostanglandin J_2_ (15d-PGJ_2_), in macrophages [18]. Additional studies from our laboratory have also demonstrated that the symptoms resulting from chemically-induced colitis, with dextran sodium sulfate (DSS), is alleviated in the colon of mice that have been supplemented with Se, characterized by increased colon length, decreased pro-inflammatory cytokines such as IL-1β, tumor necrosis factor alpha (TNFα) and interferon gamma (IFNγ) and increased anti-inflammatory markers such as arginase 1 (Arg1) [19]. Thus, Se supplementation mitigates inflammation, while increasing pro-resolutory pathways, suggesting that Se may be a potential therapeutic candidate for IBD. As such, this review will present a number of studies where the role of NF-κB and PPARγ play in the pathogenesis of IBD, the effect of Se on NF-κB and PPARγ in many cell types, and provide insight into the potential use of Se as a treatment for IBD.

## 2. NF-κB

Nuclear Factor-kappa B, a rel family of transcription factors, consisting of five subunits, namely RelA (p65), RelB, c-Rel, NF-κB1 (p105) and NF-κB2 (p100), plays an important role in a number of biologically important functions such as regulating stress response, immune responses, and inflammation [20,21]. These transcription factors can lead to the activation or repression of certain genes [21]. Under unstimulated conditions, NF-κB is inactivated and bound to members of the IκB family of proteins that prevent active NF-κB from nuclear translocation [22]. However, upon cellular stimulation through ligands such as lipopolysaccharide (LPS) and cytokines, there is a dissociation of the IκB proteins from NF-κB, through the action of the IκB kinase (IKK), consisting of the regulatory subunit NF-κB essential modulator (NEMO), IKKα (IKK1) and IKKβ (IKK2), resulting in the phosphorylation of IκB followed by its ubiquitin-dependent degradation by the proteasome. Such a dissociation of IκB from the NF-κB dimer exposes the nuclear localization sequence in NF-κB dimers to translocate into the nucleus and bind to promoters in target genes [21,23]. Under normal conditions in the gut, NF-κB activation is required for preserving the homeostatic conditions of the intestinal epithelial cells and, thus, the epithelial integrity [24]. 

It has been suggested that NF-κB is crucial for maintaining the integrity of the epithelium, but the studies conducted are somewhat contradictory. For instance, Pasparakis reported that the NF-κB signaling in the intestinal epithelium was an integral part of maintaining the homeostatic environment of the gut [25]. It was observed that mice that lack NEMO in the epithelium experienced colitis even in the absence of any form of epithelial injury. Further studies found that mice experienced increased intestinal epithelial cell death, which could act as a trigger of colitis, since the commensal bacteria could breach the epithelial barrier due to this disturbance [25,26]. However, usage of mice that lack IKK2, spontaneous colitis was not observed. It was subsequently found that this difference between the NEMO and IKK2 knock out mice was a result of the partial inhibition of the NF-κB canonical pathway in the IKK2 knock out mice, which was completely inhibited in NEMO knock out. Therefore, it is possible that there are some compensatory mechanisms in the NF-κB canonical pathway by IKK1 in IKK2 knock out mice. 

Studies have shown that there is a correlation between the levels of NF-κB in the gut and the severity of IBD. Han et al., conducted studies using Crohn’s disease patients, particularly those scheduled for resection surgery [4]. It was found that prior to surgery, histological scores conducted on colon samples revealed a correlation between NF-κB levels and histological score, where higher levels of NF-κB led to a greater histological score [4]. Currently, therapies for IBDs are aimed at blocking the NF-κB pathway [4]. Murine studies have shown that successful inhibition of NF-κB alleviates IBDs. Use of *Wasabia japonica*, a plant abundant in phytochemicals, in DSS-induced colitis inhibited the activation of NF-κB, the secretion of pro-inflammatory cytokines and prevented the onset of colitis at high dosages [27]. Similarly, Nimbolide, a phytochemical isolated from the Neem (*Azadirachta indica*) tree, alleviated both acute and chronic colitis induced in mice and inhibited NF-κB activation in macrophages [28]. 

Being a redox-sensitive transcription factor, NF-κB is also regulated by selenoproteins. Previous studies from our laboratory show that upon Se supplementation, following LPS stimulation, NF-κB activation was inhibited in macrophages through the inhibition of the phosphorylation of IκBα [29]. The ability of Se to negatively regulate NF-κB activation was dependent on the production of the arachidonic acid metabolite, 15d-PGJ_2_ [29]. The cyclopentenone moiety present in 15d-PGJ_2_ was a key factor in the inhibition of IKK2 that involved the formation of a covalent Michael reaction adduct of 15d-PGJ_2_ with essential Cys in the kinase domain of IKK2 leading to decreased kinase activity [29,30]. Studies further demonstrated that Michael electrophiles (such as 15d-PGJ_2_) could also interact with essential Cys residues in p65 and p50 to interfere in their binding to their cognate binding sites on the DNA [31]. While such a redox modulation was likely to be the underlying mechanism of Se in macrophages, other mechanisms involving the direct interaction of Se with Cys cannot be ruled out. Christensen et al. reported a decrease in NF-κB activity in prostate cancer cells, which they hypothesized could have been due to the inhibition of NF-κB through the direct interaction of Se with cysteine thiol in NF-κB [32]. Zhu et al. used Se nanoparticles coated with *Ulva lactuca* polysaccharide (ULP) to treat mice subjected with DSS-induced colitis [33]. They found that there was decreased pathology, characterized by decreased weight loss, lower disease activity index scores, and greater colon length in the mice given the Se nanoparticle compared to mice who were not given the treatment. The authors also found that there was inhibition of NF-κB activation in the mice subjected to DSS and administered the Se nanoparticle [33].

When the epithelial barrier is disturbed as in ulcerative colitis and Crohn’s Disease, immune cells become increasingly activated, resulting in the production of pro-inflammatory cytokines such as IL-6, IFNγ, and TNFα by epithelial cells, lymphocytes, and macrophages [34]. As such, NF-κB has been implicated in the etiology of IBD. Patients suffering from IBD episodes present increased activation of NF-κB produced by innate immune cells, such as macrophages, in addition to epithelial cells in the gut [34]. In fact, the activation of NF-κB in macrophages is accompanied by the production of IL-12, which is involved in the differentiation of naïve T cells into T-helper (Th1) cells [35]. It is well established that the T-cell receptor (TCR) and cluster of differentiation 28 (CD28) co-stimulation can lead to the translocation of NF-κB into the nucleus of the cell. It follows that upon stimulation of the TCR, 3-phosphoinositide–dependent protein kinase-1 (PDK1) becomes activated, leading to the formation of the CARMA1-BCL10-MALT1 complex, which activates the IKK complex leading to the subsequent activation of NF-κB [23]. It has also been shown that NF-κB modulates the activation of various CD4^+^ T cell subsets [23]. CD4^+^ T cells are key players in adaptive immune responses that is accompanied by their differentiation into Th1, Th2, and Th17 cells. While Th1 and Th2 cells are involved in host defense against intracellular pathogens and allergic and anti-parasitic responses, respectively, Th17 cells play a pivotal role in host defense against extracellular pathogens, including fungi [23,36]. Of importance to this review is that both Th1 and Th17 are key in shaping the intestinal immune responses [37,38]. 

Innate lymphoid cells (ILCs) represent another important group of immune cells localized to the mucosal surfaces and respond to the secreted molecules from the epithelium, which are involved in mounting intestinal immune responses as well as maintaining a homeostatic environment within the intestine [36,39]. ILCs are categorized into three groups based on the transcription factor that is key in their development and the cytokines produced. It has recently been shown that ILC3s can be activated in an NF-κB dependent manner, through the action of IL-18, resulting in the production of IL-22 [39]. Interestingly, both T helper cells and ILCs are implicated in the pathogenesis of IBD. It has been observed that there is an increase in the Th1 inducing cytokine, IL-12, in patients suffering from Crohn’s Disease [40]. Similarly, in ulcerative colitis and Crohn’s disease patients, an increase in the expression of the Th17 cytokine IL-17A is seen [41,42]. Patients suffering from IBDs have increased expression of the ILC3 cytokines, IL-17A and IL-22, in addition to high expression of retinoic acid receptor-related orphan receptor gamma (ROR-γt) transcription factor in the gut mucosa [43]. 

As discussed earlier, given that uncontrolled T cell activation and biased differentiation towards Th1 and Th17 cells, as well as ILC3 activation, are suggested to be one of the key causative factors in the development of IBD, the ability of Se to downregulate NF-κB activation could potentially impact such pathways. This could be effected through multiple mechanisms. First, the ability of Se to impact IL-6 and transforming growth factor beta (TGF-β) along with IL-23 could affect Th17 differentiation. IL-23 can also stimulate γδ T cells, invariant natural killer T (iNKT) cells, and intestinal innate-like sentinel T cells that promote Th17 differentiation [44,45,46]. The cytokines, TGF-β (only in murine) and IL-6 that are important for Th17 differentiation are driven by NF-κB [47,48]. IL-6 can also induce NF-κB in the intestinal epithelial cells to exert a positive loop leading to an inflammatory milieu. Secondly, the inability of NF-κB to be activated through the dissociation and degradation of IκB from the complex results in decreased Th1 activation [20]. Therefore, one can hypothesize that Se could affect this process, potentially leading to a biased response towards anti-inflammatory Th2 pathway leading to further downregulation of NF-κB activation. Finally, within the inflammatory milieu, IL-6, TNFα, and other NF-κB target genes can induce the production of PGE_2_ [49]. Increased PGE_2_ in patients with active colitis versus those in remission coupled with a reciprocal increase in PGD_2_ in the latter group suggests that switching of eicosanoid pathways from pro-inflammatory prostaglandins to anti-inflammatory bioactive lipid mediators could help resolve and sustain the remission of colitis in patients [50]. More importantly, PGE_2_ can also induce IL-23 leading to the increased Th17 differentiation. Thus, the ability of Se to resolve gastrointestinal (GI) inflammation via the decreased PGE_2_ via Th17 differentiation is an attractive hypothesis that is currently being tested in our laboratory. As ILC3s are activated by NF-κB through the action of IL-18, again, it is plausible that Se levels can potentiate the disease. Inhibiting the production of IL-18 by either dendritic cells or macrophages through Se could result in the blockade of NF-κB activation in this context. Similarly, it is probable that Se directly inhibiting the dissociation of IκB proteins from NF-κB could result in the downregulation of NF-κB, and thus, decrease the ability of NF-κB to activate ILC3 and trigger secretion of IL-17A and IL-22. 

Since oxidative stress can result in the development of IBD through destruction of the mucosal barrier of the gastrointestinal tract, it is possible selenoproteins acting as antioxidants can ameliorate the symptoms of IBD [51]. In fact, glutathione peroxidase 2 (GPx2), which is abundant in the gut, has been shown to be protective against oxidative stress during inflammation and experimental models of IBD [52,53]. SEPP1, which has both reductase and peroxidase activities, has been reported to be decreased during IBD [54]. The oxidative stress experienced during IBD can lead to the activation of NF-κB, thus selenoproteins such as GPx2 and SEPP1 can act to reduce this stress, which could lead to a decrease in the activation of NF-κB. Interestingly, thioredoxin reductase 1 (Txnrd1) can also reduce the oxidative stress during IBD as well as impact the gut microbiome through reduction of tetrathionate, which bacteria such as *Salmonella* are able to utilize as a means as outgrowing other bacteria in the gut, to thiosulfate [55]. Thus, presenting another means through which Se, through various selenoproteins can help to alleviate IBD by decreasing oxidative stress as well as diversify the microbiota.

While studies suggest a pro-inflammatory role of NF-κB, it could be argued that NF-κB is also essential for maintaining homeostasis in the intestinal epithelial cells, which could be ablated upon its inhibition. However, in the case of IBD, where NF-κB is highly activated, it is possible that Se supplementation may serve as a treatment imparting beneficial functions by decreasing NF-κB activation and creating a homeostatic environment in the gut. Needless to say, further studies are needed to ascertain if this is the case and to what extent the baseline NF-κB activity might be required for homeostasis as the ability of other transcription factors to counter NF-κB could also play an important role. 

## 3. PPARγ

Peroxisome proliferator-activated receptor gamma, PPARγ, a nuclear hormone receptor, is involved in the biosynthesis and metabolism of lipids [56,57]. PPARs, also termed lipid sensors, carry out their functions through activation by endogenous ligands that includes fatty acid metabolites [58]. PPARγ, along with other PPARs, has an anti-inflammatory function when activated by exogenous ligands, such as thiazolidinediones (rosiglitazone and pioglitazone). Of interest to this review is the ability of eicosanoids such PGD_2_ metabolites, ∆^12^-prostaglandin J_2_ (∆^12^-PGJ_2_) and 15d-PGJ_2_, which serve as endogenous ligands for PPARγ, to lead to the activation of PPARγ. Activated PPARγ, can effectively regulate downstream target genes via diverse mechanisms. Interaction of PPARγ, with its heterodimeric partner, retinoid X receptor-α (RXRα) precedes its binding to peroxisome proliferator response elements (PPREs) within the promoters of downstream target genes [59]. Another mechanism of repression of inflammatory pathways involves the ability of PPARγ: RXRα to interfere in the binding of NF-κB by a process known as “squelching” that could depend on the ability to recruit a nuclear receptor corepressor-I (NCoRI) complex that causes inhibition of expression of pro-inflammatory mediators [5,60,61]. 

Like NF-κB, PPARγ has been implicated in the regulation of colonic inflammation. In fact, it is a key receptor that is abundantly expressed in the epithelial cells of the colon, second to adipose tissue [62]. While it has been reported that there is increased expression of NF-κB during IBD, in the context of PPARγ, the opposite is observed. Interestingly, a greater decrease of PPARγ is observed in patients suffering from ulcerative colitis when compared to patients who suffer from Crohn’s disease [5]. While the majority of the information on the role of PPARγ during resolution of inflammation has been mostly restricted to macrophages, PPARγ has been demonstrated to also influence the T cell activation, in that it acts as a negative regulator of T cells following activation, through its inhibition of nuclear factor of activated T cells (NFAT), a transcription factor involved in the regulation of IL-2, which is important for T cell proliferation [63,64]. On the other hand, PPARγ can also influence regulatory T cells (T_regs_) by increasing their differentiation. 

There are several clinical and pre-clinical studies that report the mechanism underlying the role and effects of PPARγ in inflammatory disease. Dubuqouy et al., conducted studies using human subjects, where colon biopsies obtained from two patient groups revealed a decrease in PPARγ mRNA expression in both ulcerative colitis and Crohn’s disease samples, with a greater decrease in patients suffering from ulcerative colitis [65]. Interestingly, the expression of PPARγ or lack thereof was mostly confined to the epithelial cells and was correlated to the activity of ulcerative colitis [65]. Desreumaux et al., also demonstrated that there is increased susceptibility to 2,4,6-trinitrobenzenesulfonic acid (TNBS)-induced colitis in PPARγ heterozygous mice compared to their wild type counterparts [66]. Administration of a PPARγ agonist attenuated the experimental colitis. In fact, Choo et al., reported that 2-hydroxyethyl 5-chloro-4,5-didehydrojasmonate (J11-Cl), a PPARγ agonist, reduced the symptoms of DSS-induced colitis, increased the activity of PPARγ and increased the expression of anti-inflammatory cytokines [67]. Similarly, Bassaganya-Riera et al., demonstrated that conjugated linoleic acid (CLA), another PPARγ agonist, was able to induce the expression of PPARγ and inhibit TNFα expression and NF-κB activation during colitis [68]. 

Our laboratory has shown a crucial role for Se in the activation of PPARγ and its ligands, which are derived from the arachidonic acid (AA) pathway of cyclooxygenase metabolism, in macrophages. Treatment of the macrophage like Abelson leukemia virus transformed cell line (RAW264.7 macrophages) and bone marrow-derived macrophages with Se resulted in the upregulation of hematopoietic PGD2 synthase (HPGDS) and its product PGD_2_ as well as its non-enzymatic metabolites, ∆^12^-PGJ_2_ and 15d-PGJ_2_, which are agonists of PPARγ and inhibitors of NF-κB activation [18]. Activation of PPARγ by these endogenous cyclopentenone prostaglandins (CyPGs) was shown to activate the expression of HPGDS through the binding of PPARγ to the PPREs present in the proximal promoter of murine HPGDS [18]. Additionally, our laboratory has shown that Se supplementation leads to the upregulation of 15-hydroxyprostaglandin dehydrogenase (15-PGDH), the enzyme that oxidizes PGE_2_, and a subsequent decrease in PGE_2_ during DSS-induced colitis leading to the resolution of inflammation [19]. Other studies from our laboratory have shown that 15-PGDH is regulated by PPARγ, in that it can be increased by PPARγ leading to the resolution of inflammation. While the increased 15-PGDH catabolically inactivates PGE_2_, the products of PGE_2_ oxidation, 13,14-dihydro-15-keto-PGE_2_ via its dehydrated product 13,14-dihydro-15-keto-PGA_2_, could potentially increase the activation of PPARγ thus, forming a feedback loop of control of NF-κB via activation of PPARγ. This is an attractive hypothesis that remains to be completely tested. 

While there are not many studies that focus on the effect of Se and PPARγ on inflammatory bowel diseases and the mechanism through which Se acts, one can make conjectures based on the available data using murine models of IBD [67,68]. Since PPARγ is decreased in IBDs such as ulcerative colitis and Crohn’s disease and it has been shown that Se can increase both PPARγ and its ligand 15d-PGJ_2_ [5,13], it is plausible that under supplemented Se status, the disease would be significantly decreased. This effect could be mediated in a number of ways. It could be that the upregulation of PPARγ inhibits the activation of NF-κB in a number of cell types including intestinal epithelial cells, macrophages and dendritic cells, thus preventing the production of pro-inflammatory cytokines that are involved in the pathology of IBD. It has been shown that PPARγ can regulate T cell activation [63]. When activated T cells were treated with 15d-PGJ_2_ or ciglitazone, IL-2 expression was significantly inhibited [69]. Thus, it is possible that PPARγ could inhibit the differentiation of Th1 cells or the cytokine producing capabilities of these cells. Interestingly, the expression of T_regs_ during IBD is not uniform. For instance, there is decreased T_regs_ circulating in the peripheral blood of active IBD patients compared to inactive IBD patients. However, it was reported that there is increased T_regs_ in the intestinal mucosa of active IBD patients [70]. T_regs_ are defined by their expression of FoxP3, a transcription factor expressed by and that is important for the development as well as the function of T_regs_ [60]. However, T_regs_ are not the only cell type that express this, since FoxP3 is also observed on activated effector T cells [71]. Thus, activated effector T cells expressing FoxP3 could account for the increase in T_regs_ observed in the intestinal mucosa. It has been shown that PPARγ increases the expression of Foxp3^+^ T_regs_, suggesting that during IBD this could lead to greater numbers of FoxP3^+^ T_regs_ in the colon, where it is needed to suppress the actions of the activated effector T cells. Thus, Se could exert its effects through activation of PPARγ by inhibiting certain pathways or immune cell functions, which could impact cell specific functions to lead to the active resolution of inflammation in the gut. 

## 4. Complications of IBD

Complications can arise as a result of the continuous injury and repair taking place in the gut during IBD. Intestinal fibrosis, occurring in patients suffering from both ulcerative colitis and Crohn’s disease, results in strictures and obstructions in the gut [72]. The cytokine milieu of the gut during IBD can contribute to the development of intestinal fibrosis, which include, but are not limited to IL-1β, TNFα, IL-22 and IL-17. PPARγ is able to prevent the development of fibrotic cells in the gut through antagonizing a number of pathways [71]. For instance, PPARγ disrupts the transforming growth factor beta/mothers against decapentaplegic homolog 3 (TGF-β/SMAD3) pathway, which is involved in the formation of fibrotic tissues in a number of organs including the intestine, thus preventing the differentiation and activation of myofibroblasts [73,74]. It can then be speculated that Se can have an effect on intestinal fibrosis, through the actions of selenoproteins or through PPARγ. In fact, it has been reported that other forms of fibrosis such as cardiac fibrosis, resulted from a deficiency in Se. 

Colorectal cancer (CRC) is also another complication of IBD. In fact, sufferers of IBD have an increased risk of developing colorectal cancer. Clinical trials using selenium as supplementation reported a decrease in the number of colorectal cancer cases when patients administered a placebo were compared to patients administered Se [75]. Oxidative damage to DNA can result in the generation of tumors, in which case selenoproteins antioxidant properties can decrease the risk of CRC [75]. Thus, Se and selenoproteins are can be used as chemopreventative agents, especially since Se is involved in regulating apoptosis and proliferation in the intestinal epithelium. 

## 5. Conclusions

**Figure 1:**
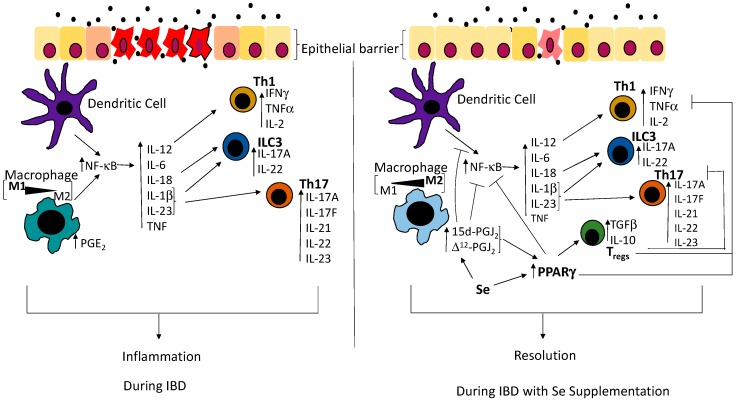
Schematic showing disruption of the epithelial barrier results in activation of immune networks that are regulated by Se. Consequently, innate immune cells such as macrophages and dendritic cells sense the presence of the bacteria and mount an immune response through the upregulation of the transcription factor, nuclear factor-kappa B (NF-κB), leading to the production of pro-inflammatory cytokines such as interleukin (IL)-18 and interleukin (IL)-12, as well as IL-1β and IL-23, which are involved in the differentiation of innate lymphoid cells (ILC3s), Th1 and Th17 cells respectively [36]. In the presence of Se, macrophages produce 15d-prostaglandin J_2_ (15d-PGJ_2_) and ∆^12^-prostaglandin J_2_ (∆^12^-PGJ_2_). The nuclear hormone receptor, peroxisome proliferator-activated receptor gamma (PPARγ), is upregulated and inhibits Th1, Th17 as well as the action of NF-κB, thus inhibiting the production of the pro-inflammatory cytokines that are required for the differentiation of T helper cells and ILC3s. Simultaneously, PPARγ leads to the differentiation of T_regs_, which inhibit the function of T helper cells. As 15d-PGJ_2_ and ∆^12^-PGJ_2_ are ligands of PPARγ, they bind to PPARγ and potentiates its action on NF-κB and T_regs_, as well as directly inhibit the activation of NF-κB. Commensal bacteria are represented by filled black circles. IFNγ: interferon gamma; TNFα: tumor necrosis factor alpha; PGE_2_: prostaglandin E_2_; IBD: Inflammatory bowel disease; IL-2: interleukin-2; IL-17A: interleukin-17A; IL-22: interleukin-22; IL-6: interleukin-6; IL-1β: interleukin-1β; IL23: interleukin-23; IL-17F: interleukin-17F; IL-21: interleukin-21; TGF-β: transforming growth factor beta; IL-10: interleukin-10.

The etiology of IBD is multifaceted. Immune responses brought about by the activation of transcription factors and nuclear receptors are implicated in the pathogenesis of the inflammatory disorders. The transcription factors NF-κB and PPARγ represent two key regulators during IBD that are differentially regulated. Interestingly, a bioactive lipid metabolite of PGD_2_ that activates PPARγ also inactivates NF-κB, in the presence of all selenoprotein expression. This review highlights areas that exploit such a unique relationship between NF-κB and PPARγ pathways, which could potentially present as an approach to mitigate inflammation while promoting sustained resolution and remission. Emerging studies point to resolution of IBD through inhibition of NF-κB and the activation of PPARγ in a number of immune cell types including macrophages, Th1, Th17 and ILC3s, where Se could play an important role (Figure 1). As discussed, it is probable that Se, through its effect on the activation of NF-κB and PPARγ, could impact the immune mechanisms to “fine tune” for effective remission. Interestingly, commensal microbiota, which can lead to IBD when the epithelial barrier is breached, can also regulate the activation of both NF-κB and PPARγ [76,77]. Studies have shown that the butyrate producing commensal *Clostridia* can activate PPARγ, while the commensal *Streptococcus salivarius* can prevent the activation of NF-κB [76,77]. It has also been reported that Se can alter the composition of the gut microbiota, which in turn can also impact the Se status of the host and consequently, the selenoproteome of the host [78]. Therefore, it appears that Se could impact commensal bacteria that regulate NF-κB and PPARγ. Furthermore, the effect of Se during an infection brought about by pathogenic bacteria could result in different immune responses when compared to the action of Se as it pertains to commensal bacteria. This review provides insights into the role of Se as a potential dietary factor that could be used as an adjunct therapy in IBD; however, further studies are needed to completely understand the intricate mechanisms the underlie the disease pathogenesis as well as the ones that follow remission.
